# A Robotic Completely Intercorporeal Jejunal Pouch Reconstruction after Gastrectomy

**DOI:** 10.3390/curroncol29110678

**Published:** 2022-11-12

**Authors:** Ani Stoyanova, Ann-Kathrin Berg, Katharina Beyer

**Affiliations:** Klink für Allgemein-und Viszeralchirurgie, Campus Benjamin Franklin, Charité—Universitätsmedizin, Hindenburgdamm Berlin 30, 12200 Berlin, Germany

**Keywords:** robot, gastrectomy, pouch, gastric cancer, Hunt-Lawrence, Pfannenstiel incision

## Abstract

Robotic surgery is increasingly gaining importance. While initial results suggest an advantage of the robotic over the minimally invasive approach in patients with gastric cancer, definitive proof of its superiority has yet to be provided. There are numerous approaches to recreate a gastric reservoir after a total gastrectomy. However, a major disadvantage of most conventional reconstructions are long term effects such as dumping syndrome, afferent loop syndrome and poor nutrition intake with severe impact on the patient quality of life. The jejunal pouch reconstruction is a beneficial reconstruction, which provides a larger reservoir capacity after gastrectomy and prevents anastomotic stenosis and dumping syndrome. The completely intercorporeal approach with a Pfannenstiel incision instead of an unfavorable midline incision can potentially decrease delayed complications such as incision hernias. With the increased deployment of robotic surgery, a complete intercorporeal reconstruction is now possible without major increase in operating time or further technical weak points. We provide for the first time a detailed technical explanation of the completely intercorporeal robotic jejunal pouch reconstruction after gastrectomy.

## 1. Introduction

Gastric cancer is one of the most common malignancies of the digestive system and the fourth leading cause of cancer-related mortality worldwide [1]. With major advances in the therapy of this cancer, more patients are eligible for curative resection. Minimally-invasive approaches are progressively dominating the gastric surgery field. Their advantages and safety even in locally advanced stages of gastric cancer could be demonstrated by numerous randomized-controlled studies [2,3,4,5,6]. With regard to robotic technology, first data from prospective randomized studies have been published comparing robot-assisted with laparoscopic gastric resection [7,8,9,10]. However, most studies so far have exclusively compared robotic versus laparoscopic distal gastrectomy [11,12]. Investigations that perform total gastrectomy comparisons in this context typically omit pouch reconstruction and are limited to laparoscopically resp. robot-assisted approaches rather than totally intercorporeal reconstructions [13]. There is solid evidence that pouch reconstruction is especially beneficial after gastrectomy with severe impact on the patient’s quality of life [14,15,16]. Thus, jejunal pouch reconstruction has been shown to provide a larger reservoir capacity after gastrectomy and decrease anastomotic stenosis and dumping syndrome [17,18]. However, when it comes to minimally-invasive surgery pouch reconstruction is still rarely performed due to the much higher demands on technical skills compared to the Roux-en-Y reconstruction.

The technique of a laparoscopically-assisted jejunal pouch reconstruction after a total gastrectomy has been described by Brenkman et al. in 2016, where he reconstruction is carried out openly via a minilaparotomy in the upper abdomen [19]. A totally intracorporeal reconstruction is technically demanding, depending on the surgeon’s acquaintance. Further, the specimen removal through Pfannenstiel incision is generally superior to laparotomy incision in regard to delayed complications such as incisional hernias [20,21]. Because of the 3D visualization and the broad angulation capabilities of the robot, facilitating surgical precision, it offers the opportunity to carry out a pouch reconstruction totally intercorporeally. Therefore, we describe systematic a robotic completely intercorporeal jejunal pouch reconstruction technique after total gastrectomy.

## 2. Materials and Methods

**Figure 1:**
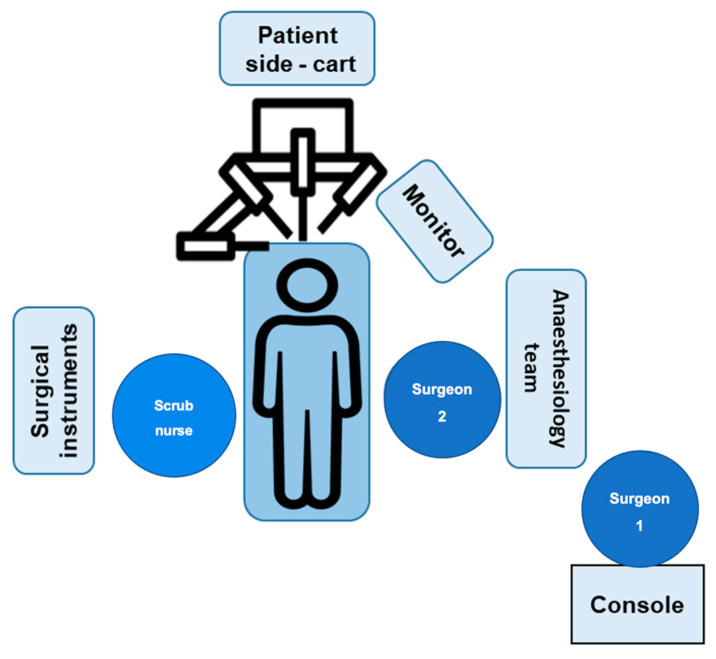
Setting description of the Da Vinci robotic system in the operating room. After the usual surgical preparations, the surgeon is seated at the da Vinci system console.

Surgery is performed using the Da Vinci X robotic surgical system (Intuitive). The robotic system consists of a console, commanded by the leading surgeon, a patient cart holding the camera and instruments, placed alongside the operating table, and a vision cart. The setting of surgical system and the positions of the involved team members are depicted in Figure 1.

The individual steps are detailed in the next paragraph. 

## 3. Procedure

### 3.1. Case Presentation

For this technical note, we present the case of a 39-year-old female patient with a signet ring cell carcinoma of the stomach, histologically diffuse type according to Laurén classification. Gastroscopy showed a flat area measuring approximetly 20 × 20 mm with a central depression in the area of the posterior wall of the stomach wtih transition to the greater gastric curvature. The patient was otherwise healthy, without further relevant morbidities. The preoperative diagnostics consisted of gastroscopy, computed tomography of the chest and abdomen, and endoscopic ultrasound. This resulted in a uT3uN+ stage with no evidence of distant metastases. According to the German S3 guideline, the patient has received four cycles of the docetaxel-based triplet chemotherapy FLOT (fluorouracil plus leucovorin, oxaliplatin and docetaxel) as a preoperative therapy. Restaging was then performed using gastroscopy and computed tomography of the thorax and abdomen showing a moderate response of the local findings to the chemotherapy. There was still no evidence of distant metastases. The patient was admitted the day of surgery. The surgical procedure is described step-by-step in the following sections. The surgical time comprised 4 h 18 min. Intraoperative frozen sections revealed negative proximal and distal resection margins. In total, 38 lymph nodes were removed with the specimen. The patient was cardiopulmonary stable and did not require monitoring in the ICU ward. The mobilization and food intake were tolerated well and the patient was discharged on the 7th postoperative day.

### 3.2. Port Trocar Placement and Intraabdominal Inspection

**Figure 2:**
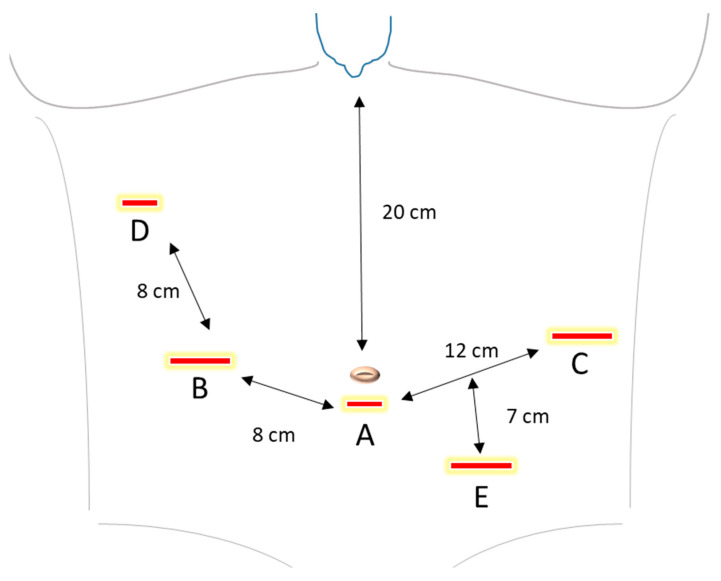
Trocar placement. A. Camera port (8 mm). B. 1. Robotic arm (12 mm). C. 2. Robotic arm (12 mm). D. 3. Robotic arm (8 mm). E. Assistant trocar (12 mm). The recommended distance between the port trocars is 8 cm between A and B, as well as B and D, and 12 cm between A and C. The incision for the optic can be done above or under the navel, depending on the patient’s height and torso length. The recommended distance from the camera trocar to the sternum is 20 cm. At least 2 cm distance from the bottom edge of the rib cage is recommended.

After the general anesthesia, the patient is placed in supine position, with both arms straight against the torso. The trocar setup is specified in Figure 2. After performing appropriate safety tests according to the manufacturer’s instructions, the Verres needle is inserted, followed by application of CO_2_ to establish pneumoperitoneum. The intraabdominal pressure is maintained at 12 mmHg. An optical trocar is inserted through a 9 mm incision above or under the umbilicus, depending on the depending on the patient’s height and torso length. A thorough laparoscopic inspection of the intraabdominal cavity is performed to detect any signs of peritoneal carcinosis, ascites, adhesions, liver metastasis or further pathologies. Two 8 mm trocars as well as two 12 mm trocars are placed as described in Figure 2. The patient is placed in a 30 degrees anti-Trendelenburg position. Then, the camera and instrument arms of the daVinci robot are docked. The system alignment setup is checked for optimal camera position and to avoid potential instrument collisions.

### 3.3. Preparation of the Greater Omentum

**Figure 3:**
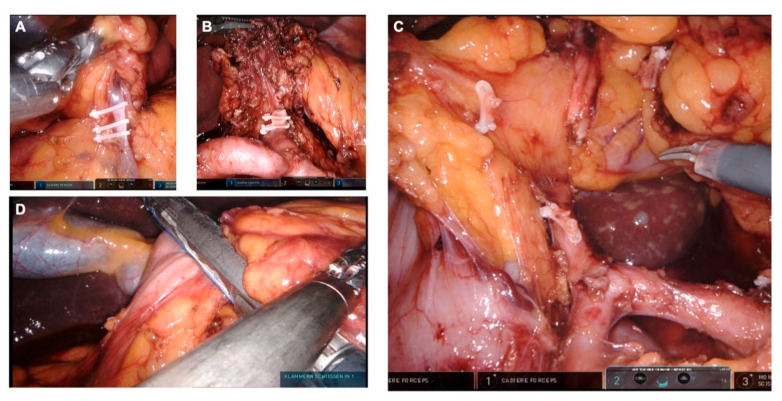
Lymphadenectomy. (**A**) Exposure of the right gastroepiploic vein. (**B**) Exposure of the celiac trunc, the common hepatic artery, the splenic artery. The left gastric artery has been divided. (**C**) Exposure of the gastroduodenal artery, the common hepatic artery and the proper hepatic artery. The right gastric artery has been divided. (**D**) Closure of the duodenal stump.

The greater omentum is detached from the transverse colon. We begin with the dissection to the left patient site, exposing the left Arteria and Vena gastroepiploica. These vessels are removed directly at their origin from the splenic vessels, using endoscopic ligation Hem-o-lok^®^ clip applier. In the process, the surrounding lymphatic tissue is added to the preparation. Then, we proceed with further dissection with division of the short gastric vessels proximate to the spleen. This removes and leaves the lymph node stations 4e and 4s en bloc on the specimen. Next, the fundus is mobilized, so that the left diaphragmatic crus can be exposed. We proceed with the depiction of the splenic artery and lymphadenectomy along the splenic artery. The lymph node stations 10 and 11 are removed. We continue with the dissection of the greater omentum to the right patient site. The right colon flexure is mobilized, so that the duodenum can be exposed. The right gastroepiploic artery and vein are exposed and discontinued at the lower edge of the pancreas using Hem-o-Lok^®^ clips (Figure 3A). With that, the removal of the lymph node station 6 is complete. The lymphatic tissue on the gastroduodenal artery is then dissected and left on the specimen.

### 3.4. Suprapyloric Preparation

While the stomach is further lifted with the robotic arm 4, the upper edge of the pancreas is exposed and the peritoneum at the upper edge of the pancreas is incised. First, we dissect at the level behind the hepatic artery and expose the portal vein. Here the left gastric vein can be exposed and divided between Clips. Then the lymphatic tissue around the hepatic artery is dissected and left on the specimen. With this exposure, the lymphadenectomy is performed up to the celiac trunk and along the A. hepatica propria. The right gastric artery is exposed and transected between clips (Figure 3C). With this, lymph node station 5 is dissected. In addition, the hepatic bifurcation is exposed and the lymphatic tissue is dissected. After the presence of an aberrant hepatic artery has been ruled out, an incision of the pars flaccida of the lesser omentum is performed, followed by dissection of the right crus and exposure of the abdominal esophagus. Next, we expose the left gastric artery as well as the celiac trunk in this order and perform a lymphadenectomy along the vessels. Thus, the lymph node stations 7 and 9 are dissected. Then we proceed with the exposure of the splenic artery and completion of the lymphadenectomy of station 11. In doing so, the preparation planes can be combined. The duodenum is transected about 2 cm distal to the pylorus and divided using an endoscopic stapler (Figure 3D). Then the left gastric artery is transected (Figure 3B). The vessel stump is secured with two Hem-o-lok^®^ clips. The cardia is then closed with a ligature and the esophagus is cut off in its abdominal part. Then the specimen is placed in an extraction bag.

The specimen can now be removed through a Pfannenstiel incision, after the insertion of an Alexis wound retractor. If the frozen section procedure shows tumor-free oral and distal resection margins, the laparoscopy can be resumed to verify completeness of the lymphadenectomy and hemostasis.

### 3.5. Cholecystectomy

We proceed with a cholecystectomy. Therefore, Calot’s triangle with the cystic artery and Ductus cysticus are exposed. After occlusion of the cystic artery and cystic duct between Lapro-Clips, the gallbladder is detached from the liver bed and removed.

### 3.6. Jejunal Pouch Reconstruction

**Figure 4:**
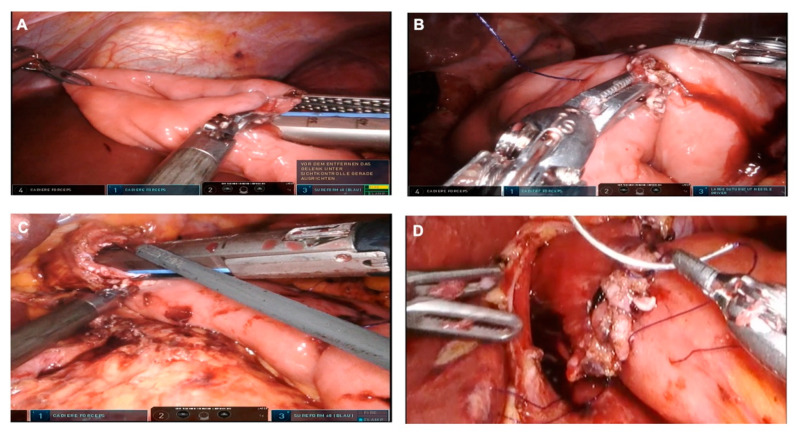
Jejunal pouch reconstruction. (**A**) A jejunal pouch is produced using an endoscopic stapler, two to three cartridges are usually required. (**B**) Closure of the enterotomy at the aboral (distal) end of the pouch. (**C**) Creation of the posterior wall of the esophagojejunostomy using a linear stapler. (**D**) The anterior wall of the esophagojejunostomie is created by means of barbed sutures.

**Figure 5:**
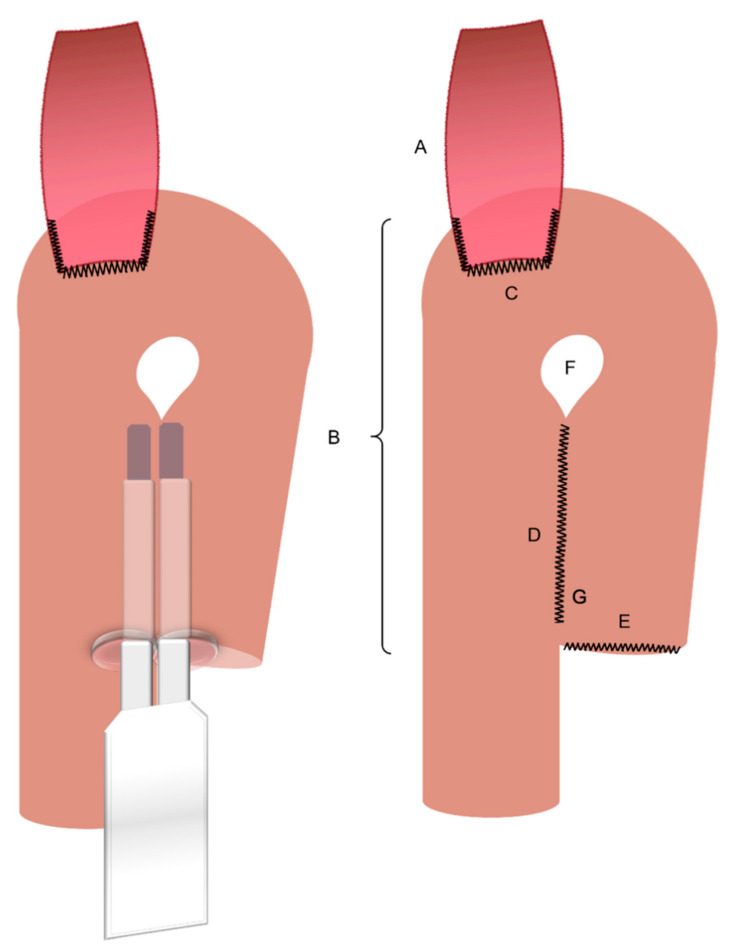
Jejunal pouch reconstruction—model; A. Esophagus. B. Jejunal pouch (~15 cm). C. Esophagojejunostomy (modified collard anastomosis). D. Jejunal plication. E. Jejunal stump. F. Intentional incomplete stapling of the jejunal plication. G. Barbed suturing of the stapler insertion area.

First, we expose the ligament of Treitz and identify a 20 cm long section of the jejunum distal from the ligament of Treitz. The mesenterium is dissected in an avascular area and the jejunum is divided using an endoscopic stapler. We proceed with a preparation of a non-vascularized area of the transverse mesocolon. The alimentary loop of the jejunum is passed through the preparated window in the mesocolon in order to connect the distal esophageal end. Then a jejunal pouch with a length of about 15 cm is produced using an endoscopic stapler. The oral bridge, on which the future anastomosis will be located, is not severed. The enterotomy at the end of the pouch is closed using barbed sutures. We proceed with an antimesenteric incision just below the apex of the jejunal pouch. The posterior wall of the esophagojejunostomy is fabricated by inserting an endoscopic stapler into the incision in the pouch and into the exposed esophageal stump to create a common lumen. The anterior wall of the esophagojejunostomy is sutured continuously using two barbed sutures. A leak test is performed by instilling lipofundin via gastric tube to prove a primary sufficient anastomosis. The base anastomosis with the bile-carrying loop of the reconstruction is created about 45 cm distal to the Hunt-Lawrence esophagojejunostomy as a side-to-side jejunojejunostomy (functional end-to-end anastomosis) using a 60 mm endoscopic stapler. The staple insertion area is closed using barbed suture. Finally, this results in a Y-Roux Hunt-Lawrence pouch reconstruction (Figure 4 and Figure 5). A drain is placed at the anastomosis. The daVinci robot is undocked. The trocars are removed under vision, the pneumoperitoneum is let off and the abdomen is closed.

### 3.7. Postoperative Management

The nasogastric tube is removed on the first postoperative day and the patient is allowed to sip up to 600 mL water per day. On the second postoperative day, patients start receiving enteral feeding, whereby the nutritional intake is being slowly increased, supplemented with parenteral nutrition. In our current experience, patients are usually discharged by 7–10 days after the surgery.

## 4. Discussion

This technical description provides for the first time a comprehensive guidance to a robotic completely intercorporeal jejunal pouch reconstruction after total gastrectomy. Our current experience in the surgical and postoperative clinical management indicates that this method of pouch reconstruction is safe and does not lead to major prolongation of the operating time.

There are numerous approaches to recreate a gastric reservoir after a total gastrectomy in patients with gastric cancer. However, a major disadvantage of most conventional reconstructions are long term effects such as dumping syndrome, afferent loop syndrome and poor nutrition intake with severe impact on the patient quality of life. A jejunal pouch reconstruction after gastrectomy has been previously described [19]. The advantages of the jejunal pouch are remarkable and have been repeatedly attested [16]. The pouch provides a larger reservoir capacity after gastrectomy, and has the potential to reduce the occurrence of dumping syndrome by significantly slowing down the emptying time [22]. A textbook size of the pouch has not been clearly defined to this day. Previous studies show that a length of the pouch between 15 to 20 cm leads to less reflux symptoms, a shorter pouch reduces the eating capacity, whereby a longer pouch could potentially disrupt the emptying process [23,24]. For this method, we reconstruct a pouch, which has a length of about 15 cm.

As described in this technical note, we are performing an incomplete stapling of the pouch. However, the precise technique of the jejunal pouch reconstruction is still to be established. The question of whether the jejunal plication should be extended up to the esophagojejunostomy has been discussed among surgeons over the past few decades. Halabi and Lawrence appointed the impaired blood flow in the area of the anastomosis as the reason for esophagojejunostomy leakage, which was attributed to jejunal plication reaching the anastomosis. Therefore, they suggested an incomplete stapling of the proximal pouch site. The result was the reconstruction known today as the “Hunt-Lawrence-Pouch” (Figure 5) [25]. Regardless, there is currently no solid evidence that the Lawrence reconstruction is superior to a complete plication of both jejunal limbs.

Another intriguing debate is regarding the simultaneous gallbladder removal during gastrectomy. In the controlled-randomized study CHOLEGAS, cholecystectomy did not increase morbidity, following the procedure. While cholecystectomy was effective in preventing long-term occurrence of gallstones or sludge, these have rarely occurred or mostly remained asymptomatic in patients who did not receive removal of the gallbladder. The symptoms of gallstones, if they occur at all, are mostly delayed, therefore particularly patients with a longer life expectancy could benefit from the procedure. In view of these data, we perform cholecystectomy in younger patients, or in patients with early tumor stages or pre-existing gallbladder stones as part of the gastrectomy [26].

There are numerous publications suggesting an advantage of minimally invasive compared to open gastrectomy [2,3,4,5,6]. This also applies to locally advanced stages, which are prevalent in the western world [2]. However, the technically less demanding distal resections predominate in the available studies [2]. Additionally, in most studies investigating total gastrectomy, the reconstruction is performed according to Roux en Y employing a small midline incision [2]. In view of the current evidence, neither a midline incision [20,21] nor a reconstruction without pouch [16] can be considered the optimal procedure. Thus, incisional hernias have rarely been reported in Pfannenstiel incisions compared to the midline laparotomy [27].

One reason why little experience with minimally invasive pouch reconstruction is reported in the literature could be the high technical demands of this operation. The technical challenges of this operation increase even more when the reconstruction is performed completely intercorporeally. However, a complete intercorporal reconstruction is necessary for retrieving the specimen via a Pfannenstiel incision instead of a median laparotomy. The robot could offer an advantage here with its flexible joints and excellent three-dimensional visualization.

In this regard, human error in surgery can obviously be reduced by optimizing the working environment and cater for an ultimate comfort for the surgeon. A clear benefit of the robotic surgical system is that it generally requires less physical effort when compared to laparoscopic surgery [28]. The lightning eliminates the need of an oftentimes heavy surgical head light. The three-dimensional vision improves the overview with enlarged organ presentation and high-definition of small vessels and nerves. Undeniably, these conditions allow maximum surgical control and precision.

All of the above discussed benefits of the robotic surgery and the described method of the completely intercorporeal pouch reconstruction after gastrectomy could not be realistically achieved without the main actor—a particularly specialized and experienced upper gastrointestinal surgeon. Structured training concepts, certification by reliable providers, and especially long-term and extensive training in the field of gastric surgery at a lower-level complexity are the main requirements for this method. However, the excitement around robotic surgery should be moderate considering several factors. The colossal price of a surgical robotic system limits the adoption of this approach to the leading clinical centers. An exhaustive surgical training for the robot use to an advanced level also comes with an impressive price-tag. In practice, on a local level the use of the robotic system is oftentimes, and understandably, reserved for the chief of the clinic or mostly some of the leading attending surgeons. Because of this relatively young surgical approach, “the passing of the knowledge” to the unfamiliarized fellows is still quite restricted. With the increased deployment of robotic surgery, we are optimistic that this approach will be more approachable in the coming days. And with this technical note, we present that a complete intercorporeal jejunal pouch reconstruction after gastrectomy is possible without major increase in operating time or further technical weak points.

## 5. Conclusions

We provide a comprehensive presentation of the robotic intercorporeal jejunal pouch reconstruction after gastrectomy. This completely intercorporeal approach can potentially decrease long-term complications such as incision hernias in addition to the positive impact on the patient’s quality of life due to the pouch reconstruction after gastrectomy. However, this procedure requires the acquaintance of a trained surgeon with an extensive expertise in the laparoscopic field. We are confident that our detailed depiction of the jejunal pouch reconstruction will provide a solid basis for future advances in the surgical gastrectomy practice.

## Data Availability

Not applicable.
